# A colorimetric method for the molecular weight determination of polyethylene glycol using gold nanoparticles

**DOI:** 10.1186/1556-276X-8-538

**Published:** 2013-12-20

**Authors:** Kai Ling, Hongyan Jiang, Qiqing Zhang

**Affiliations:** 1 Key Laboratory of Biomedical Material of Tianjin, Institute of Biomedical Engineering, Chinese Academy of Medical Sciences & Peking Union Medical College, Tianjin 300192, People’s Republic of China

**Keywords:** Gold nanoparticles, Polyethylene glycol, Molecular weight determination, Colorimetric method, Spectrophotometry

## Abstract

A gold nanoparticle (AuNP)-based colorimetric method was developed for the molecular weight (MW) determination of polyethylene glycol (PEG), a commonly used hydrophilic polymer. Addition of a salt solution to PEG-coated AuNP solutions helps in screening the electrostatic repulsion between nanoparticles and generating a color change of the solutions from wine red to blue in 10 min in accordance with the MW of PEG, which illustrates the different stability degrees (SDs) of the AuNPs. The SDs are calculated by the absorbance ratios of the stable to the aggregated AuNPs in the solution. The root mean square end-to-end length (〈*h*^2^〉^1/2^) of PEG molecules shows a linear fit to the SDs of the PEG-coated AuNPs in a range of 1.938 ± 0.156 to 10.151 ± 0.176 nm. According to the Derjaguin-Landau-Verwey-Overbeek theory, the reason for this linear relationship is that the thickness of the PEG adlayer is roughly equivalent to the 〈*h*^2^〉^1/2^ of the PEG molecules in solution, which determines the SDs of the AuNPs. Subsequently, the MW of the PEG can be obtained from its 〈*h*^2^〉^1/2^ using a mathematical relationship between 〈*h*^2^〉^1/2^ and MW of PEG molecule. Applying this approach, we determined the 〈*h*^2^〉^1/2^ and the MW of four PEG samples according to their absorbance values from the ordinary ultraviolet–visible spectrophotometric measurements. Therefore, the MW of PEG can be distinguished straightforwardly by visual inspection and determined by spectrophotometry. This novel approach is simple, rapid, and sensitive.

## Background

Polyethylene glycol (PEG) is a synthetic hydrophilic polymer, which is widely used as an emulsifier and surfactant in cosmetics, foodstuffs, and pharmaceutical products [1,2]. The molecular weight (MW) of PEG has a significant impact on its properties and applications [1,3,4]. In the case of PEG-functionalized drugs, in particular, an increase in the MW of PEG leads to reduced kidney excretion, resulting in a prolonged blood circulation time of the drug [1]. A variety of analytical techniques, such as size exclusion chromatography (SEC) with preferably a universal detector [2], nuclear magnetic resonance spectroscopy [5], and matrix-assisted laser desorption ionization time-of-flight mass spectrometry [6], have been used to determine the MW of PEG polymer. However, these powerful techniques require the use of sophisticated instruments and complicated protocols. Besides, the instruments are not as readily available in many laboratories.

Gold nanoparticle (AuNP)-based colorimetric assays have attracted considerable attentions in detection applications with regard to their simplicity and versatility [7,8]. This colorimetric assay can be easily observed by visual inspection, which avoids the relative complexity inherent in conventional detection methodologies [9]. Because of the electrostatic repulsion resulting from the negative charges on the surfaces, AuNPs are highly stable in the absence of added salts. The addition of electrolytes to gold sols results in the reduction of charge repulsion and as a consequence nanoparticle aggregation. Nonetheless, AuNPs can be stabilized even at high salt concentrations by adsorbing proteins or other hydrophilic polymers (protecting agents) onto their surfaces [10]. They bind the macromolecules by noncovalent electrostatic, stable adsorption [11]. PEG polymer is one of the most often used stabilizers, as it possesses the advantage of a chemically well-defined composition that ensures the reproducibility of its performance. Moreover, PEG dissolves rapidly and therefore can be prepared just prior to use.

At high salt concentrations, the stability of PEG-coated AuNPs depends upon the MW of PEG [12]. The stabilization of the fully coated AuNPs is due to the steric repulsion effect, which is dependent on the thickness (*t*) of the PEG adlayer and the conformation of the adsorbed PEG molecules [10,13,14]. The adsorbed PEG forms a single protecting layer on the surface of the nanoparticle, because of the resistance of the polymer coil to compress and to release both bound and free water from within the hydrated coil [15-17]. Under the complete coverage of the surface condition, PEG molecules are in direct competition for the adsorption sites on the AuNP surface [18]. Therefore, the adsorbed linear PEG molecules form typical loops and tail conformations [13,18]. The value of *t* is roughly equivalent to the size of the PEG molecule as a free molecule in solution under the condition [13,18]. The root mean square end-to-end length (〈*h*^2^〉^1/2^) is commonly used to specify the size of a linear polymer molecule.

Herein, enlightened by the above facts, we developed a simple and reliable colorimetric method for the MW determination of PEG in aqueous solution using citrate-reduced AuNPs. This method is based on the different stability degrees (SDs) of the AuNPs, which are fully coated by different MW (〈*h*^2^〉^1/2^) of PEG, after screening the electrostatic repulsion between nanoparticles. The SDs of the AuNPs are monitored by ultraviolet–visible (UV–vis) spectrophotometry, which exploits the strong sensitivity of the localized surface plasmon resonance spectrum to the aggregation of AuNPs. In this study, the SDs are calculated by the absorbance ratios of the stable to the aggregated AuNPs in solution. The nanoparticles exhibit greater stability upon an increase in the MW (〈*h*^2^〉^1/2^) of PEG. Of the systems tested, the 〈*h*^2^〉^1/2^ of PEG molecules was found to exhibit a good linear correlation to the SDs of the AuNPs in a specified range. As a result, we can obtain the 〈*h*^2^〉^1/2^ of PEG from the SDs of the AuNPs and then estimate the corresponding MW using a mathematical relationship between the 〈*h*^2^〉^1/2^ and MW of PEG molecule. So far, there is no report on nanomaterial-based methods for the MW determination of polymers. This AuNP-based determination method offers simplicity, convenience, and sensitivity, and can be accomplished in minutes without sophisticated instruments or training overhead.

## Methods

### Materials

Hydrogen tetrachloroaurate (III) trihydrate (HAuCl_4_ · 3H_2_O) and four PEG samples (SPEG 1,450 to 10,000) were purchased from Sigma-Aldrich (St. Louis, MO, USA). Ten PEG samples (APEG 400 to 20,000) were purchased from Alfa Aesar (Tianjin, China). Trisodium citrate dihydrate (Na_3_C_6_H_5_O_7_ · 2H_2_O), sodium azide (NaN_3_), and sodium chloride (NaCl) were purchased from Sinopharm Group Chemical Reagent Co., Ltd. (Shanghai, China). All chemicals were analytical grade reagents and used without further purification. All water was deionized by reverse osmosis and further purified using a Milli-Q Plus system (Millipore, Billerica, MA, USA) to 18.2 MΩ cm resistivity. All glassware were cleaned using aqua regia solution (HCl/HNO_3_ = 3:1, *v*/*v*) and subsequently rinsed with a copious amount of Milli-Q treated water.

### AuNP preparation

Citrate-reduced AuNPs were prepared according to the modified method [19,20]. In brief, 99.00 mL of water and 1.00 mL of 1.0% (*w*/*v*) HAuCl_4_ · 3H_2_O solution were mixed in a flask. The mixture was then heated under magnetic stirring until it began to boil, and a 1.0% (*w*/*v*) Na_3_C_6_H_5_O_7_ · 2H_2_O solution (1.80 and 2.25 mL) was quickly added to the solution. After boiling for 20 min, the solutions were cooled to room temperature (25°C) with vigorous magnetic stirring. The prepared AuNP solutions were stored at 4°C until ready for use. The nanoparticle concentrations of the prepared two samples were determined by measuring their extinction at 520 and 524 nm, respectively.

The prepared nanoparticles were characterized using a JEM-2010 FEF transmission electron microscope (TEM; JEOL Ltd., Akishima, Tokyo, Japan). Bright-field images of at least 200 particles deposited onto a carbon-coated copper grid (Xinxing Braim Technology Co., Ltd., Beijing, China) were measured using ImageTool graphics software to approximate the average particle diameter. The optical densities of the two AuNP samples at 520 and 524 nm, respectively, were measured using a Lambda 35 UV–vis spectrophotometer (Perkin Elmer, Waltham, MA, USA).

### Colorimetric determination of PEG MW

Fully PEG-coated AuNPs were formed by the addition of 3-mL PEG solution (15 mg/mL) to 1 mL of the as-prepared AuNP solution. Immediately after adding the PEG solution, the suspension was ultrasonicated (KQ-100DY, Kun Shan Ultrasonic Instruments Co., Ltd., Jiangsu, China) for 10 min and then incubated over 16 h with gentle agitation using an orbital shaker at low speed (<1 Hz) to allow the polymer to adsorb to the nanoparticles. The PEG-coated nanoparticles were collected by centrifugation (12,000 rpm, 20 min) and resuspended in water three times to wash out the free PEG molecules and produce the fully coated AuNPs used in subsequent examinations. Subsequently, 1-mL aliquots of PEG-coated AuNP solutions were mixed with a certain volume (40, 50, or 60 μL) of 10.0% (*w*/*v*) NaCl solution at room temperature (25°C) for 30 s, followed by recording of their absorption spectra using the Lambda 35 UV–vis spectrophotometer after 10 min.

### Chromatographic determination of PEG MW

SEC measurements were performed using a Waters 515 liquid chromatography system configured with an Optilab rEX refractive index (RI) detector (Wyatt Technology, Santa Barbara, CA, USA). Separations were performed using three size exclusion columns (SB804HQ, SB803HQ, and SB802.5HQ, Shodex, Japan) in series. PEG samples (100 μL) were run at 5 mg/mL concentrations in aqueous solution. The running buffer contained 0.05% (*w*/*v*) NaN_3_. A flow rate of 0.5 mL/min was used, and samples were characterized using RI detection (internal temperature 30°C). The columns and the buffers were used at the same temperature.

Multi-angle laser light scattering (MALLS) measurements were used to perform analytical scale chromatographic separations for the absolute MW determination of the principal peaks in the above SEC/RI measurements. MALLS determinations were performed using an 18-angle DAWN HELEOS laser light scattering detector (Wyatt Technology, USA) connected in tandem to the Optilab rEX RI detector (Wyatt Technology, USA), operating with a 50-mW solid-state laser at 658 nm. System and instrument validation was performed based on dextran (GPC Standard 80, Pharmacosmos, Denmark).

### Dynamic light scattering measurements

Hydrodynamic radii (*R*_h_) of PEG molecules were measured by dynamic light scattering (DLS) (Nanosizer ZS, Malvern Instruments, Worcestershire, UK) at room temperature (25°C). All PEG samples were dissolved in 81.5 mM NaCl solution to 5 mg/mL concentrations. All PEG solutions were then ultrasonicated for 10 min and filtered through 0.22-μm nylon filters. The zeta potentials of the AuNPs were also measured by DLS at room temperature (25°C).

### Data analysis

OriginPro 8.0 software (OriginLab, Northampton, MA, USA) was employed to perform data processing. Each sample measurement was repeated in triplicate, and the data were presented as the mean ± standard deviation.

## Results and discussion

Colloidal nanoparticles in a dispersion medium always show Brownian motion and hence undergo frequent collisions with each other. The stability of colloids is thus determined by the interaction between the nanoparticles during such collisions. There are two basic interactions: one being attractive and the other repulsive. When attraction dominates, the nanoparticles will aggregate with each other, and finally, the entire dispersion may coalesce. Conversely, when repulsion dominates, the system will be stable and remain in a dispersed state. This idea was originally proposed by Derjaguin, Landau, Verwey, and Overbeek and is therefore referred as the DLVO theory [13,21]. The DLVO theory assumes that the behavior of colloidal nanoparticles can be simplified by the interaction potential between two neighboring nanoparticles [13,21].

We therefore used the DLVO theory to study the effects of PEG MW on the stability of the coated AuNPs. The three major interaction energies at work in this system are electrostatic (*U*_elec_) and steric (*U*_steric_) repulsions and van der Waals (*U*_vdW_) attraction. These are assumed to be additive so that the total interparticle interaction energy (*U*_total_) becomes [22]

(1) $$U_{\mathrm{total}}=U_{\mathrm{elec}}+U_{\mathrm{steric}}+U_{\mathrm{vdW}}.$$

**Figure 1:**
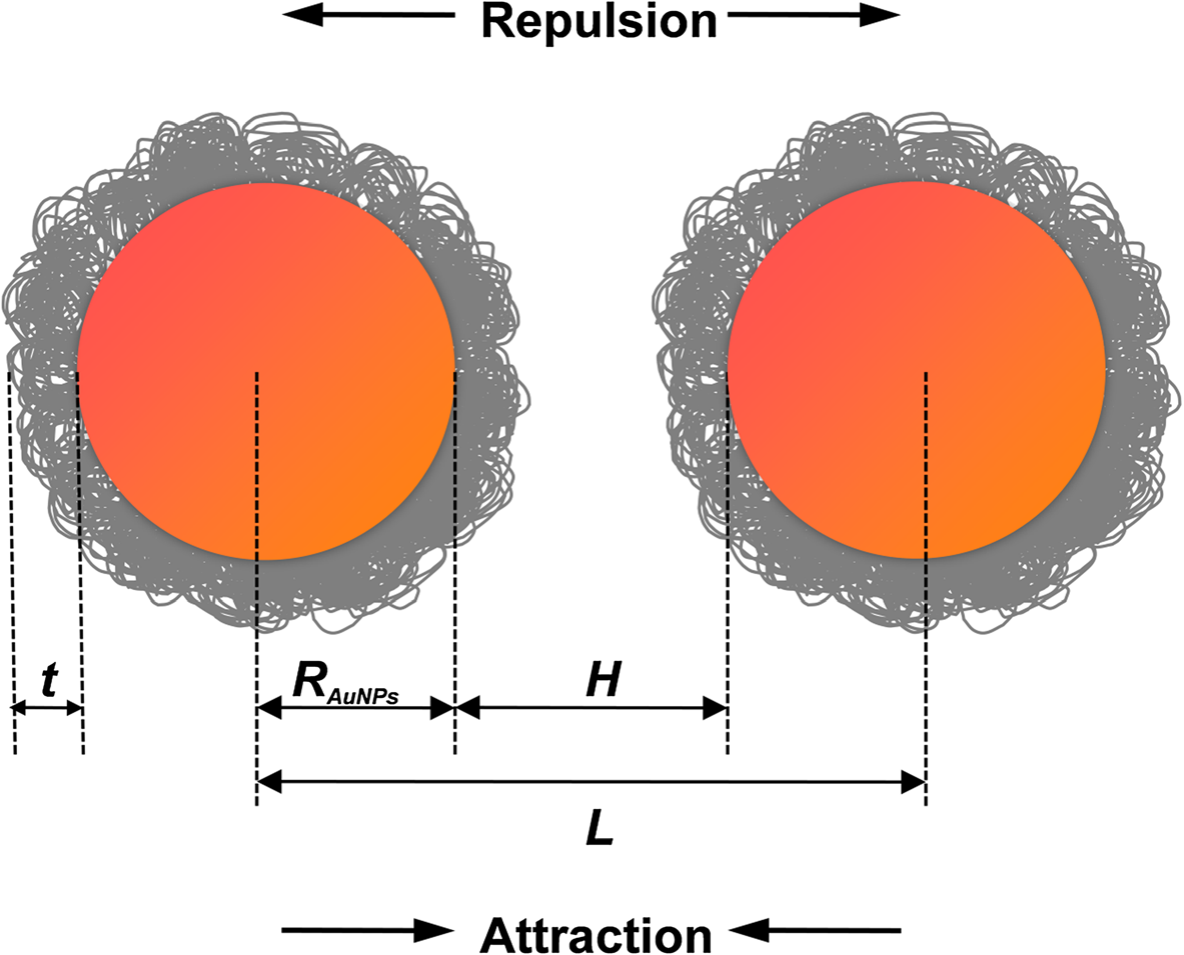
**Schematic of two neighboring AuNPs coated with adsorbed PEG. ***R*_AuNPs_ is the radius of the AuNPs, *L* is the nanoparticle center-to-center separation distance, *H* is the separation distance between the nanoparticle surface (*H* = *L* − 2*R*_AuNPs_), and *t* is the thickness of the adsorbed PEG layer.

We estimated the interaction energies for two neighboring spherical AuNPs coated by PEG adlayer as shown in Figure 1.

The weight average molecular weights (*M*_w_) and the *R*_h_ of the PEG samples determined from the above experiments are shown in Table 1. The polydispersity indexes (*M*_w_/*M*_n_) of all PEG samples were measured to be about 1.05. Due to the conformation of hydrated PEG molecules (low *M*_w_) in aqueous solution, the radii of gyration (*R*_g_) of the PEG coils can be calculated by [23]

(2) $$R_{h}=0.85R_{g}.$$

**Table 1:** ***M***_**w**_**, *R***_**h**_**, *R***_**g **_**(Equation** 2**), and 〈** ***h***^**2**^**〉**^**1/2 **^**(Equation** 3**) values of PEG samples used in this study**

**Samples**	***M***_**w **_**(Da)**^**a**^	***R***_**h **_**(nm)**^**b**^	***R***_**g **_**(nm)**^**c**^	**〈** ***h***^**2**^**〉**^**1/2 **^**(nm)**^**d**^
APEG 400	378 ± 30	0.568 ± 0.027	0.668 ± 0.032	1.636 ± 0.078
APEG 600	521 ± 51	0.672 ± 0.054	0.791 ± 0.064	1.938 ± 0.156
APEG 1,000	997 ± 77	0.944 ± 0.025	1.111 ± 0.029	2.721 ± 0.072
APEG 2,000	1,887 ± 20	1.602 ± 0.284	1.885 ± 0.334	4.617 ± 0.818
APEG 4,000	3,981 ± 82	1.784 ± 0.165	2.099 ± 0.194	5.141 ± 0.475
APEG 6,000	6,185 ± 165	2.343 ± 0.111	2.756 ± 0.131	6.751 ± 0.320
APEG 8,000	8,232 ± 162	2.749 ± 0.101	3.234 ± 0.119	7.922 ± 0.291
APEG 10,000	10,535 ± 907	3.306 ± 0.063	3.889 ± 0.074	9.526 ± 0.182
APEG 12,000	13,646 ± 1359	3.522 ± 0.061	4.144 ± 0.072	10.151 ± 0.176
APEG 20,000	19,118 ± 631	4.415 ± 0.015	5.194 ± 0.018	12.723 ± 0.043
SPEG 1,450	1,348 ± 64	1.203 ± 0.097	1.415 ± 0.114	3.466 ± 0.280
SPEG 4,600	4,384 ± 436	2.095 ± 0.045	2.465 ± 0.053	6.038 ± 0.130
SPEG 8,000	8,350 ± 301	2.572 ± 0.299	3.026 ± 0.352	7.412 ± 0.862
SPEG 10,000	10,641 ± 219	3.474 ± 0.214	4.087 ± 0.252	10.011 ± 0.617

^a^*M*_w_ was determined by MALLS. ^b^*R*_h_ was determined by DLS. ^c^*R*_g_ was calculated using Equation 2. ^d^〈*h*^2^〉^1/2^ was calculated using Equation 3.

Since the PEG chains behave much like ideal chains in water, the *R*_g_ is related to the 〈*h*^2^〉^1/2^, which is expressed by the following equation [23,24]:

(3) $${\langle h^{2}\rangle }^{1/2}=\sqrt{6}R_{g}.$$

The data of the above calculations are listed in Table 1.

According to the previous reports, a relationship exists between the *M*_w_ and the *R*_g_ of PEG, and a linear fit of these variables yields the coefficient *υ* with the relationship *R*_g_ ∝ *M*_w_^*υ*^[23-25]. Moreover, when the *M*_w_ is low (<80,000 Da), the effects of excluded volume interactions diminish, and *υ* → 0.5 [23,25,26]. When *υ* = 0.5, a polymer chain behaves in an ideal (Gaussian) manner in a *θ* solvent [23]. Since the 〈*h*^2^〉^1/2^ is directly proportional to the *R*_g_ (Equation 3), 〈*h*^2^〉^1/2^ ∝ *M*_w_^*υ*^[24], which is described by

(4) $${\langle h^{2}\rangle }^{1/2}=0.0718M_{w}^{0.5250}$$

with an *R*^2^ = 0.9994. This relationship is presented in Additional file 1: Figure S1 and plotted according to the *M*_w_ and the 〈*h*^2^〉^1/2^ values of the PEG samples (APEG 400 to 20,000) listed in Table 1. The coefficient *υ* is 0.5250, which is close to 0.5, establishing the fact that the PEG chains behave much like ideal chains in the solution [23].

In order to verify the colorimetric method, two sizes of AuNPs were prepared by reducing HAuCl_4_ with different amounts of trisodium citrate (see ‘Methods’). Through TEM examination, the diameters of the as-prepared AuNPs were measured to be about 16 and 26 nm, respectively (Additional file 1: Figure S2). The zeta potential values of the AuNPs were measured to be −34.6 ± 1.9 mV (16-nm AuNPs) and −30.1 ± 1.5 mV (26-nm AuNPs) by DLS. The nanoparticle concentrations were calculated as 1.316 × 10^−9^ M (16-nm AuNPs) and 2.804 × 10^−10^ M (26-nm AuNPs) using the corresponding absorbance values of 0.6471 and 0.6911 at 520 and 524 nm in conjunction with the calculated extinction coefficient for $\epsilon_{\mathrm{NP}16}^{520\;\mathrm{nm}}=4.916\times 10^{8}$ and $\epsilon_{\mathrm{NP}26}^{524\;\mathrm{nm}}=2.465\times 10^{9}$ cm^−1^ M^−1^, respectively [27].

To ensure that the amounts of PEG are able to saturate the AuNP surfaces in the final suspensions of 7.925 × 10^11^ particles/mL (16-nm AuNPs) and 1.689 × 10^11^ particles/mL (26-nm AuNPs), we estimated the total surface area simply based on the diameters of the uncoated AuNPs. Thus, the total available surface area in the suspensions was estimated as approximately 6.37 × 10^−4^ m^2^/mL (16-nm AuNPs) and 3.59 × 10^−4^ m^2^/mL (26-nm AuNPs). We then calculated the amount of PEG needed to cover all nanoparticles with a single monolayer of four typical PEG samples (APEG 400, 600, 6,000, and 20,000) occupying areas dictated by their *R*_h_ (Additional file 1: Tables S1 and S2). These numbers were then compared to the total concentration of PEG available in the solution for the bulk concentration used (11.25 mg/mL). This concentration is considered to ensure that there are at least 5 orders of magnitude more PEG molecules than necessary as needed to saturate the nanoparticle surfaces, based on the above calculations.

The Debye length (*κ*^−1^) is the measure of a charge carrier's net electrostatic effect in the solution and the distance over which those electrostatic effects persist. It is also appropriately termed the electrostatic ‘screening length,’ beyond which the charges are electrically screened [13]. For a single symmetrical electrolyte in water at room temperature (25°C), it can be readily calculated in the form [13]:

(5) $$\kappa^{-1}=\frac{0.3041}{\left|z\right|\sqrt{C}}\left(\mathrm{nm}\right),$$

where *C* is the electrolyte concentration (M) and *z* is the valence of the electrolyte.

In this study, we added varying amounts of 10.0% NaCl solution (40, 50, or 60 μL, *w*/*v*) to each PEG-coated AuNP solution (1 mL) to screen the electrostatic repulsion between nanoparticles. The electrostatic repulsion originates from the surface underlying the adsorbed polymer layer. The resulting NaCl concentrations were 65.8, 81.5, and 96.9 mM, respectively. The corresponding values of *κ*^−1^ were determined to be 1.19, 1.07, and 0.98 nm, which were calculated using the above data and Equation 5. The amount of the salt present in the added 40 μL of 10.0% (*w*/*v*) NaCl solution does not ensure complete screening of the electrostatic repulsion. This may be attributed to the fact that the *R*_h_ of APEG 400 is 0.568 nm (2*R*_h_ < *κ*^−1^ = 1.19 nm) and the zeta potentials of the fully coated nanoparticles range from −13.4 (APEG 400, 16-nm AuNPs) to −9.5 mV (APEG 20,000, 16-nm AuNPs) and from −12.6 (APEG 400, 26-nm AuNPs) to −8.4 mV (APEG 20,000, 26-nm AuNPs) after adding NaCl solution. The salt added in a 50-μL amount of 10.0% (*w*/*v*) NaCl solution can more adequately screen the electrostatic repulsion as a result of the relatively shorter *κ*^−1^ with the zeta potentials ranging from −8.3 (APEG 400, 16-nm AuNPs) to −4.8 mV (APEG 20,000, 16-nm AuNPs) and from −7.8 (APEG 400, 26-nm AuNPs) to −4.4 mV (APEG 20,000, 26-nm AuNPs) after NaCl addition. Likewise, the amount of salt for the addition of 60 μL of 10.0% (*w*/*v*) NaCl solution can also screen the electrostatic repulsion. However, the hydrophobicity of soluble polymer increases at a higher concentration of salt [28,29]. Hence, 50 μL of 10.0% (*w*/*v*) NaCl solution was added to 1 mL of PEG-coated AuNP solutions in order to screen the electrostatic repulsion between nanoparticles. In addition, the pH values of the PEG-coated AuNP solutions were maintained at 6.3, even after salt addition. According to the above analyses, the *U*_elec_ = 0, under the salt addition condition.

The steric repulsion between two nanoparticles of radius *R*_AuNPs_ with adsorbed PEG layers can be modeled as [30]

(6) $$U_{\mathrm{steric}}=\begin{array}{c} \left\{\begin{array}{c} \infty ;L<2R_{\mathrm{AuNPs}}\\ U_{0}\left[-\ln\left(y\right)-\frac{9}{5}\left(1-y\right)+\frac{1}{3}\left(1-y^{3}\right)-\frac{1}{30}\left(1-y^{6}\right)\right];2R_{\mathrm{AuNPs}}\\ \;0;L>2\left(R_{\mathrm{AuNPs}}+t\right) \end{array}\right)\\ \;<L<2\left(R_{\mathrm{AuNPs}}+t\right), \end{array}$$

where

(7) $$y=\frac{L-2R_{\mathrm{AuNPs}}}{2t}\leq 1$$

and

(8) $$U_{0}=\left(\frac{\pi^{3}t\sigma_{p}k_{B}T}{12N_{p}l^{2}}\right)R_{\mathrm{AuNPs}}t^{2},$$

where *L* is the radial distance from the center of particles, *σ*_*p*_ is the surface density of adsorbed chains, *k*_B_ is the Boltzmann constant, *T* is the kinetic temperature, *N*_p_ is the number of segments in the polymer chain, and *l* is the segment length.

The potential energy of the van der Waals interaction between two particles, *U*_vdW_, can be approximated by the following calculation [14],[21]:

(9) $$U_{\mathrm{vdW}}=-\frac{A^{*}R_{\mathrm{AuNPs}}}{12H},$$

where A^*^ is the effective Hamaker constant and *H* is the separation distance between the surfaces of the core particles. According to the DLVO theory, when the surface layers just touch (i.e., *H* = 2 *t*), the *U*_steric_ = 0. The total energy (*U*_total_) of the net interaction has a deep minimum that is dependent on the value of the *U*_vdW_ (Additional file 1: Figure S3) [13,18,31]. In general, the minimum of the *U*_total_(dashed line in Additional file 1: Figure S3) determines the stability of fully coated AuNPs, which is dependent on the *t* value of the adlayer [13]. If the adlayer is thick enough, the minimum becomes so slight that it can be ignored, thus resulting in greater nanoparticle stability, and vice versa [13]. In other words, the *t* can determine the SDs of the PEG-coated AuNPs.

**Figure 2:**
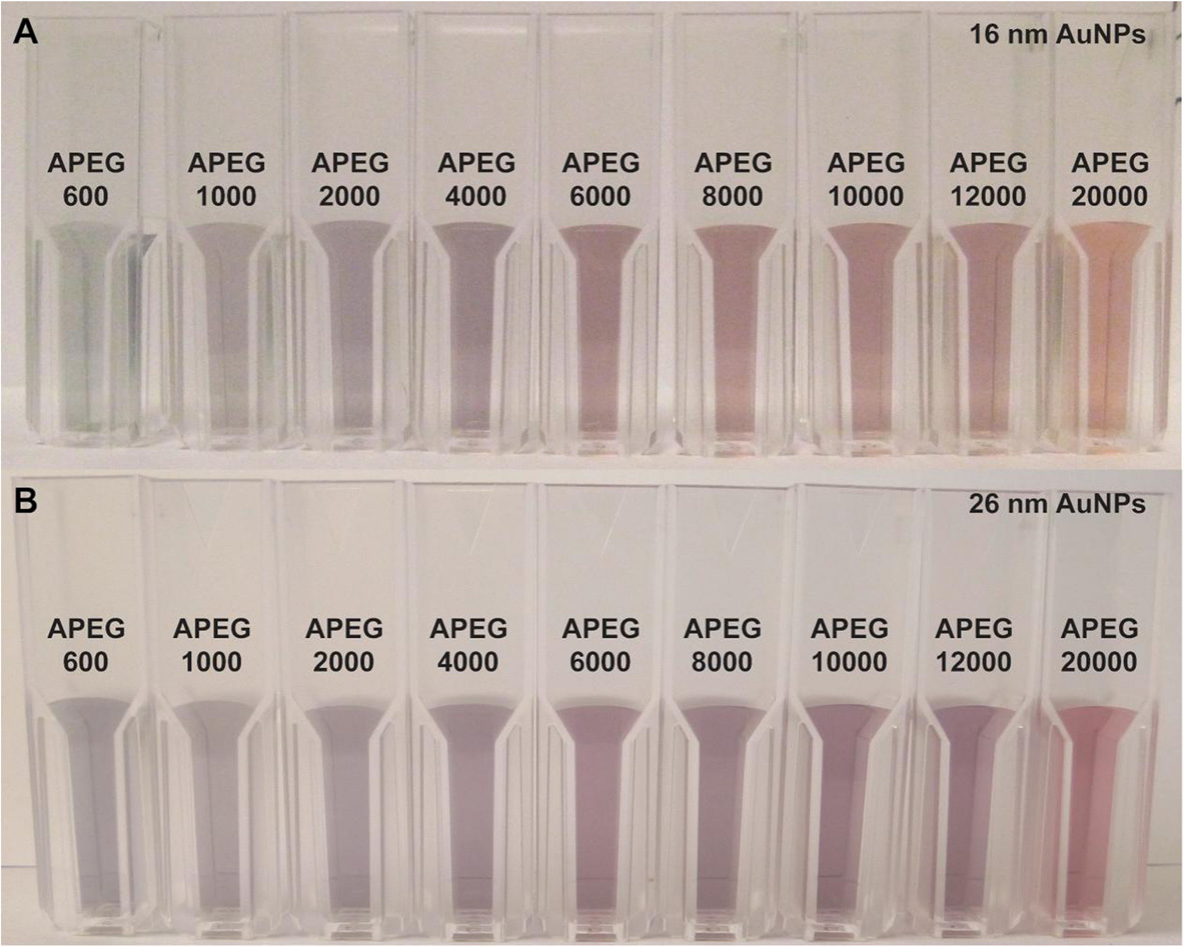
**Visual color change of AuNPs coated with adsorbed PEG of different MW. (A)** 16-nm AuNPs and **(B)** 26-nm AuNPs.

**Figure 3:**
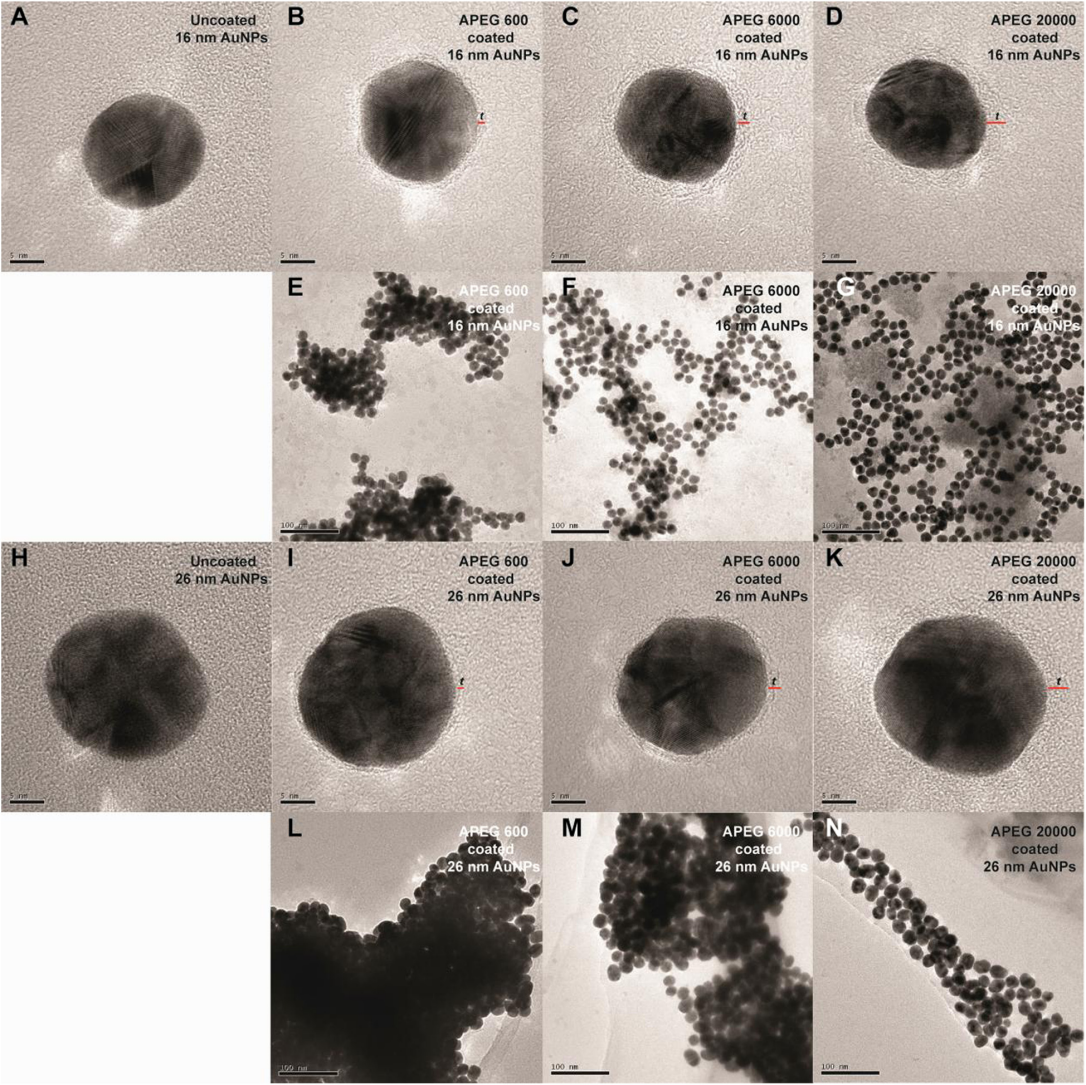
**TEM images of uncoated and PEG-coated AuNPs.** TEM images of uncoated AuNPs: **(A)** 16-nm AuNPs and **(H)** 26-nm AuNPs. TEM images of fully coated AuNPs in the absence of 10.0% (*w*/*v*) NaCl solution for 16-nm AuNPs: **(B)** APEG 600, **(C)** APEG 6,000, and **(D)** APEG 20,000; for 26-nm AuNPs: **(I)** APEG 600, **(J)** APEG 6,000, and **(K)** APEG 20,000. TEM images of fully coated AuNPs in the presence of 10.0% (*w*/*v*) NaCl solution for 16-nm AuNPs: **(E)** APEG 600, **(F)** APEG 6,000, and **(G)** APEG 20,000; and for 26-nm AuNPs: **(L)** APEG 600, **(M)** APEG 6,000, and **(N)** APEG 20,000. The *t* represents the thickness of the dehydrated PEG adlayer (red line). The scale bars are 5 nm **(A to D)**, **(H to K)** and 100 nm **(E to G)**, **(L to N)**, respectively.

After screening the electrostatic repulsion, the colors of the PEG-coated AuNP solutions were observed to change from wine red to blue within 10 min of NaCl addition, in accordance with the MW of PEG (Figure 2). The APEG 400-coated AuNPs aggregated rapidly to form a deposit within 3 to 5 min, so the data are not shown. However, the APEG 20,000-coated AuNPs remained stable, without significant aggregation (color change) during the experimental period (8 h). This phenomenon reflects the differences in the SDs of the AuNPs. This color change supports the ready distinction of PEG MW through visual inspection. TEM was employed to examine the PEG adlayers on the typical fully coated nanoparticle surfaces (by APEG 600, 6,000, and 20,000). As shown in Figure 3, higher MW of PEG corresponded to a thicker adlayer, and hence, greater AuNPs stability.

**Figure 4:**
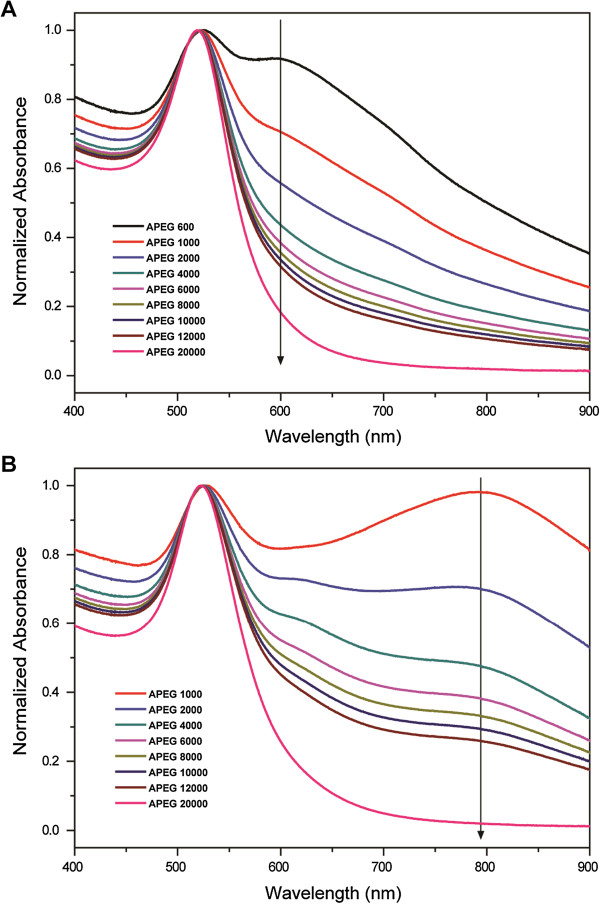
**Normalized absorption spectra of PEG-coated AuNPs in the presence of 10.0% (*w*/*v*) NaCl solution. (A)** 16-nm AuNPs and **(B)** 26-nm AuNPs.

Figure 4 shows the normalized absorption spectra of the PEG-coated 16- and 26-nm AuNPs in 81.5 mM NaCl solution. The absorption peaks at 520 nm (16-nm AuNPs) and 524 nm (26-nm AuNPs) are attributed to the still stable nanoparticles in the solution. The other absorption peaks at 598 nm (16-nm AuNPs) and 790 nm (26-nm AuNPs) correspond to the aggregated nanoparticles in the solution. In this study, we used the absorbance ratios of the stable to the aggregated nanoparticles in the solution to calculate the SDs of the AuNPs, which are formulated by

(10) $$SD_{\left(16\;\mathrm{nm}\right)}=\frac{A_{520}}{A_{598}-A_{598}^{0}}$$

(11) $$SD_{\left(26\;\mathrm{nm}\right)}=\frac{A_{524}}{A_{790}-A_{790}^{0}}$$

where, the $A_{598}^{0}$ and the $A_{790}^{0}$ are the absorbance values of the diluted AuNP solutions (1 mL of PEG-coated AuNP solution + 50 μL of water) at 598 nm (16-nm AuNPs) and 790 nm (26-nm AuNPs), respectively. The APEG 600-coated 26-nm AuNPs began to form a precipitate within 10 min, and hence, the data are not shown.

**Figure 5:**
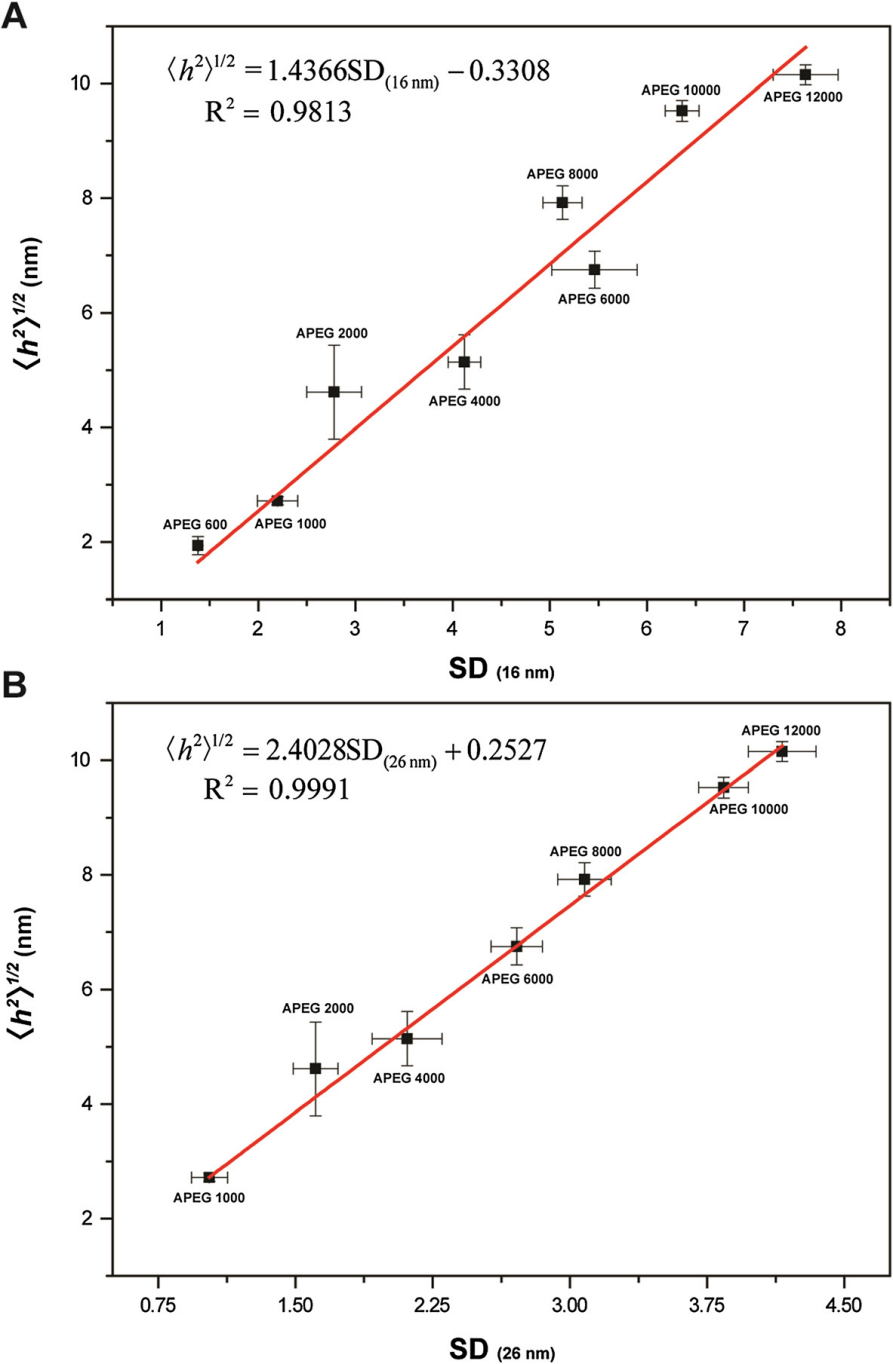
**Linear correlation between the 〈*h***^**2**^**〉**^**1/2 **^**of PEG and the SDs of fully coated AuNPs. (A)** 16-nm AuNPs and **(B)** 26-nm AuNPs.

In this study, the 〈*h*^2^〉^1/2^ values of PEG were found to exhibit a good linear correlation to the SDs of the fully coated AuNPs in the range of 1.938 ± 0.156 nm (APEG 600) to 10.151 ± 0.176 nm (APEG 12,000, Figure 5). The reason is attributed to the *t* of the PEG adlayer being about equal to the 〈*h*^2^〉^1/2^ of the PEG molecules in solution under the system condition [13,18]. For PEG-coated 16-nm AuNPs (APEG 600 to 12,000), the standard regression equation is

(12) $${\langle h^{2}\rangle }^{1/2}=1.4366SD_{\left(16\;\mathrm{nm}\right)}-0.3308$$

with an *R*^2^ = 0.9813. For PEG-coated 26-nm AuNPs (APEG 1,000 to 12,000), the standard regression equation is

(13) $${\langle h^{2}\rangle }^{1/2}=2.4028SD_{\left(26\;\mathrm{nm}\right)}+0.2527$$

with an *R*^2^ = 0.9991. Therefore, the 〈*h*^2^〉^1/2^ of PEG can be estimated through the absorbance values of UV–vis spectrophotometric measurements. Finally, the *M*_w_ of PEG can be obtained using Equation 4.

The colorimetric method was employed to determine the 〈*h*^2^〉^1/2^ of SPEG samples. The normalized absorption spectra of the AuNPs coated with SPEG 1,450, 4,600, 8,000, and 10,000 in the NaCl solution are presented in Additional file 1: Figure S4. According to their absorbance values, the 〈*h*^2^〉^1/2^ values of the four PEG samples are estimated through Equations 12 and 13. Then, using Equation 4, the *M*_w_ of the PEG is obtained from its calculated 〈*h*^2^〉^1/2^. The above results are listed in Table 2. The measurements obtained by this colorimetric method did not exhibit a significant difference compared to the SEC/MALLS method with the two-tailed Student's *t* test (*P* > 0.1).

**Table 2:** **AuNP-based colorimetric method to determine 〈** ***h***^**2**^**〉**^**1/2 **^**and *M***_**w **_**values of PEG samples**

**Samples**	**16-nm AuNPs**		**26-nm AuNPs**	
	**〈** ***h***^**2**^**〉**^**1/2 **^**(nm)**	***M***_**w **_**(Da)**	**〈** ***h***^**2**^**〉**^**1/2 **^**(nm)**	***M***_**w **_**(Da)**
SPEG 1,450	3.398 ± 0.298	1,561 ± 259	3.444 ± 0.411	1,611 ± 362
SPEG 4,600	6.017 ± 0.368	4,621 ± 537	6.096 ± 0.349	4,736 ± 515
SPEG 8,000	8.086 ± 0.279	8,096 ± 532	7.974 ± 0.397	7,893 ± 747
SPEG 10,000	9.903 ± 0.432	11,919 ± 989	10.032 ± 0.387	12,212 ± 897

## Conclusions

In summary, a unique colorimetric method was developed to determine the MW of PEG, based on the steric stabilization of PEG-coated AuNPs. Using the ordinary UV–vis spectrophotometry technique, the MW of the PEG samples can be calculated by the absorbance values of the PEG-coated AuNP solutions, after adding salt to screen the electrostatic repulsion between nanoparticles. This strategy offers operational advantages (simplicity, convenience, and sensitivity) over many existing methodologies, which has important implications for the development of nanomaterial-based determination methods. In the future, this colorimetric method can be applied to the MW determination of other soluble macromolecules. This strategy would provide a great advantage to current research areas in polymer science, materials science, and biology.

## Abbreviations

APEG: PEG samples were purchased from Alfa Aesar; AuNPs: Gold nanoparticles; DLS: Dynamic light scattering; MALLS: Multi-angle laser light scattering; Mw: Weight average molecular weights; MW: Molecular weight; PEG: Polyethylene glycol; RI: Refractive index; Rh: Hydrodynamic radii; Rg: Radii of gyration; SD: Stability degree; SEC: Size exclusion chromatography; SPEG: PEG samples were purchased from Sigma-Aldrich; TEM: Transmission electron microscope; UV–vis: Ultraviolet–visible; κ−1: Debye length; 〈h2〉1/2: Root mean square end-to-end length.

## Competing interest

The authors declare that they have no competing interests.

## Authors’ contributions

KL and HJ performed the experiments and analyzed the results. QZ conceived and designed the experiments, analyzed the results, and participated in writing the manuscript. All authors read and approved the final manuscript.

## Authors’ information

KL and HJ are Ph.D. holders, and QZ is a professor. All authors are from the Key Laboratory of Biomedical Material of Tianjin, Institute of Biomedical Engineering, Chinese Academy of Medical Sciences & Peking Union Medical College, Tianjin 300192, People's Republic of China.

## Supplementary Material

Additional file 1 **Supplementary information of a colorimetric method for the molecular weight determination of polyethylene glycol.** Correlation between 〈*h*^2^〉^1/2^ and *M*_w_ of PEG **(Figure S1)**. TEM images of as-prepared AuNPs **(Figure S2)**. Plot of energy vs interparticular distance (*H*) for steric stabilization **(Figure S3)**. Normalized absorption spectra of PEG (SPEG 1,450 to 10,000)-coated AuNPs in the presence of 10.0% (*w*/*v*) NaCl solution **(Figure S4)**. Calculation of surface area of 16-nm AuNP availability for PEG adsorption **(Table S1)**. Calculation of surface area of 26-nm AuNP availability for PEG adsorption **(Table S2)**. Click here for file
