# Supplementary material for: A colorimetric method for the molecular weight determination of polyethylene glycol using gold nanoparticles
Source: Nanoscale Res Lett. 2013 Dec 20;8(1):538. doi: 10.1186/1556-276X-8-538 (PMC3878192; doi:10.1186/1556-276X-8-538)
Supplement: Additional file 1 — Supplementary information of a colorimetric method for the molecular weight determination of polyethylene glycol. Correlation between 〈h2〉1/2 and Mw of PEG (Figure S1). TEM images of as-prepared AuNPs (Figure S2). Plot of energy vs interparticular distance (H) for steric stabilization (Figure S3). Normalized absorption spectra of PEG (SPEG 1,450 to 10,000)-coated AuNPs in the presence of 10.0% (w/v) NaCl solution (Figure S4). Calculation of surface area of 16-nm AuNP availability for PEG adsorption (Table S1). Calculation of surface area of 26-nm AuNP availability for PEG adsorption (Table S2). [file 1556-276X-8-538-S1.pdf]

Supplementary Information

A colorimetric method for the molecular weight determination of polyethylene glycol using gold nanoparticles

Kai Ling, Hongyan Jiang, Qiqing Zhang*

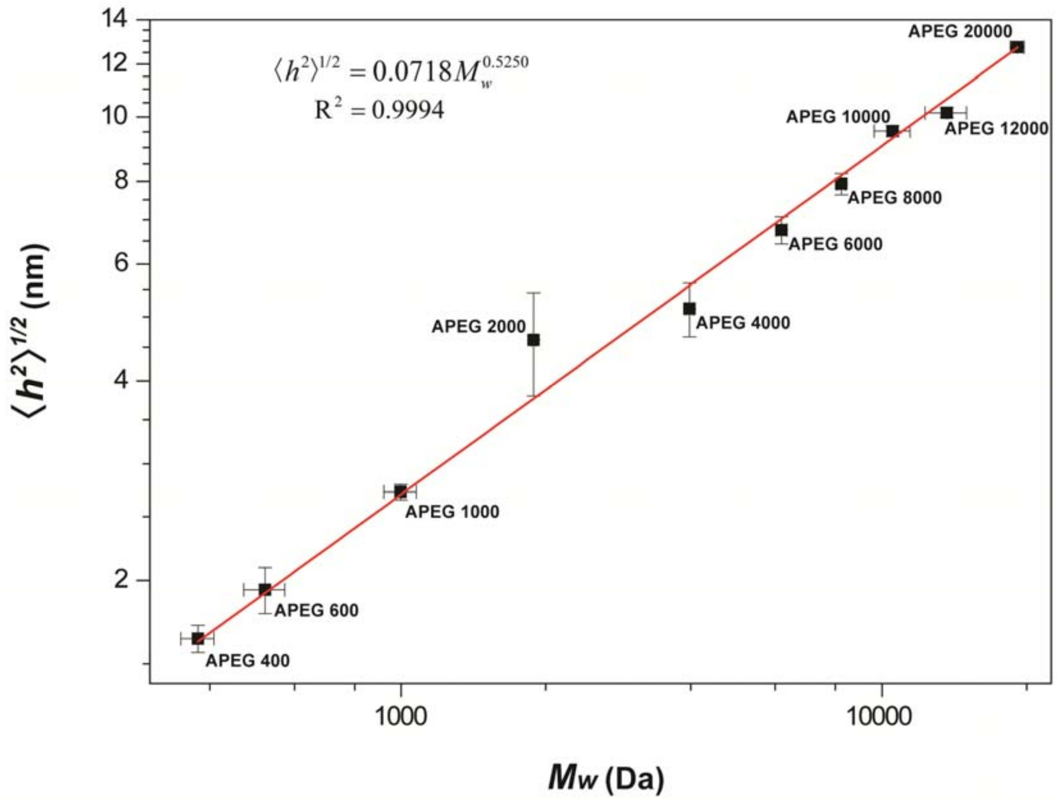

Figure S1 Correlation between $\langle h^2\rangle^{1/2}$ and M_w of PEG (APEG 400 –20000, log scale).

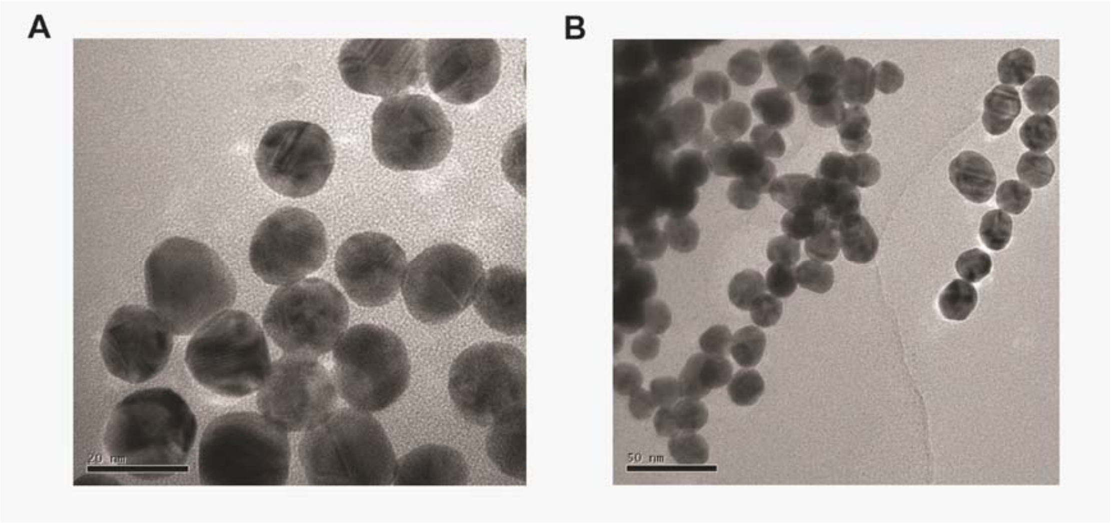

Figure S2 TEM images of as-prepared AuNPs. (A) 16 nm AuNPs, the scale bar is 20 nm; (B) 26 nm AuNPs, the scale bar is 50 nm.

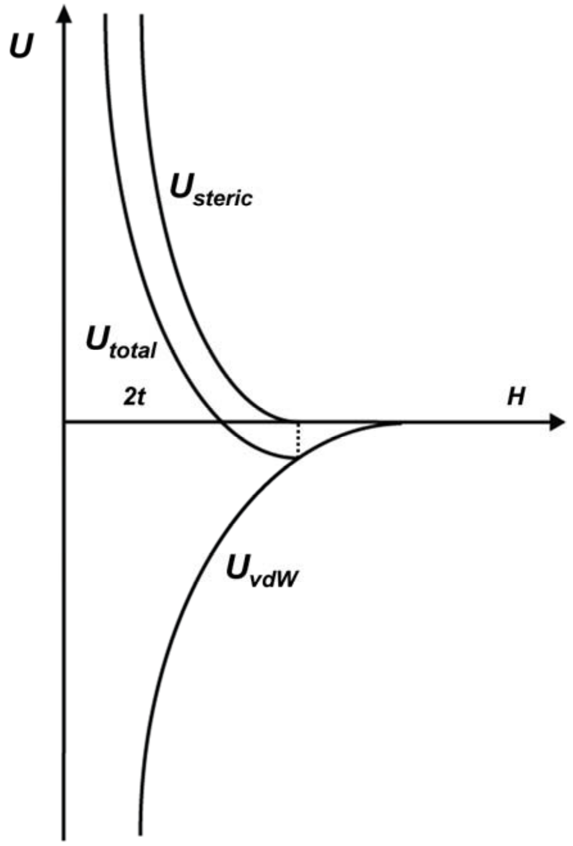

Figure S3 Plot of energy vs interparticular distance (*H*) for steric stabilization.

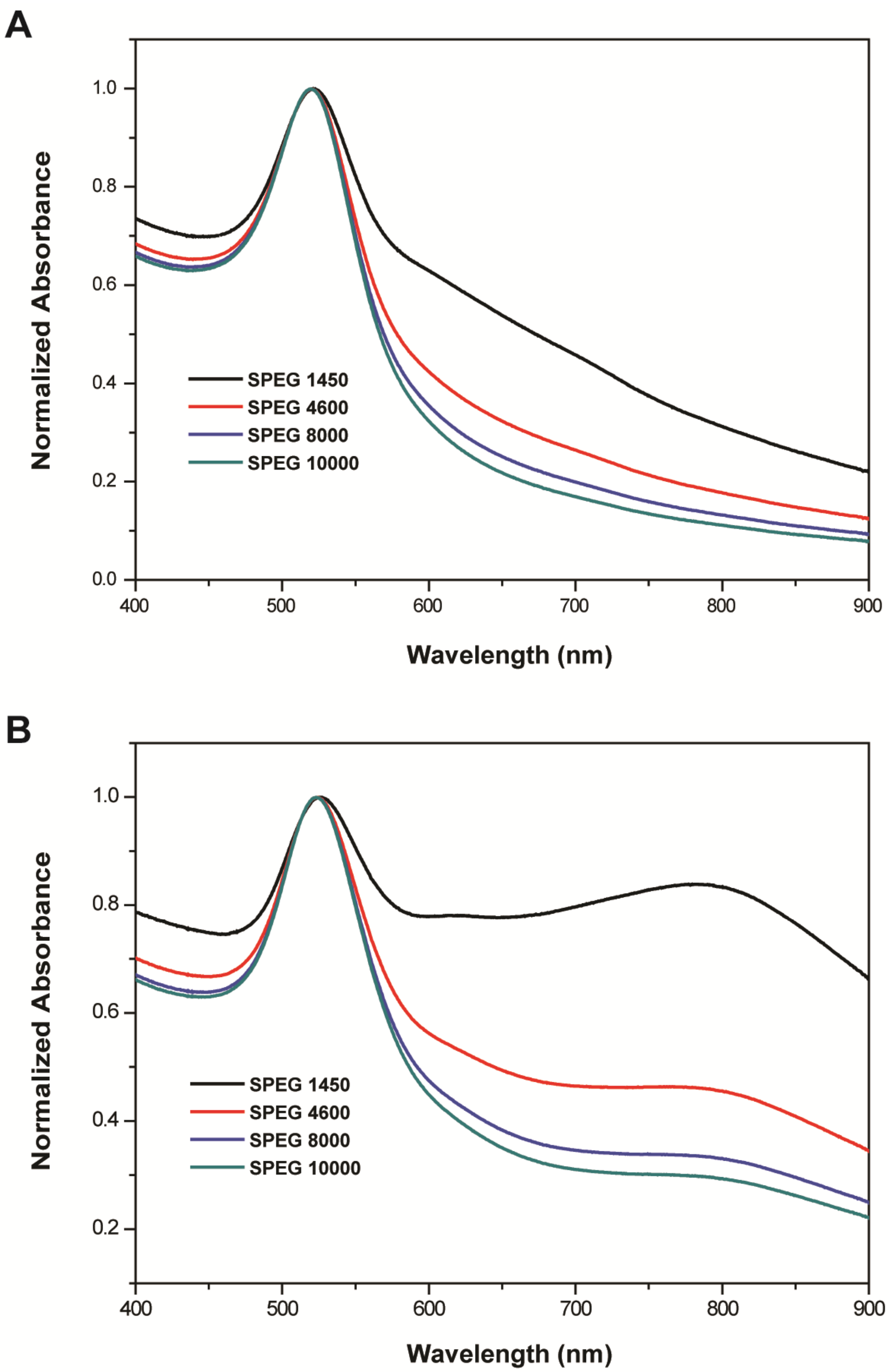

Figure S4 Normalized absorption spectra of PEG (SPEG 1450 – 10000) -coated AuNPs in the presence of 10.0% (w/v) NaCl solution. (A) 16 nm AuNPs, (B) 26 nm AuNPs.

Table S1 Calculation of surface area of 16 nm AuNPs availability for PEG adsorption

Samples	M_w (Da)	R_h (nm)	Available molecules (10^{17}/mL)	Molecules needed to form single layer (10^{12}/mL)	Order of magnitude excess
APEG 400	378 ± 30	0.568 ± 0.027	180.37 ± 14.32	158.19 ± 15.01	5
APEG 600	521 ± 51	0.672 ± 0.054	131.29 ± 12.85	114.45 ± 18.28	5
APEG 6000	6185 ± 165	2.343 ± 0.111	10.96 ± 0.29	9.30 ± 0.88	5
APEG 20000	19118 ± 631	4.415 ± 0.015	3.55 ± 0.12	2.60 ± 0.02	5

Table S2 Calculation of surface area of 26 nm AuNPs availability for PEG adsorption

Samples	M_w (Da)	R_h (nm)	Available molecules (10^{17}/mL)	Molecules needed to form single layer (10^{12}/mL)	Order of magnitude excess
APEG 400	378 ± 30	0.568 ± 0.027	180.37 ± 14.32	89.16 ± 8.46	5
APEG 600	521 ± 51	0.672 ± 0.054	131.29 ± 12.85	64.50 ± 10.30	5
APEG 6000	6185 ± 165	2.343 ± 0.111	10.96 ± 0.29	5.24 ± 0.50	5
APEG 20000	19118 ± 631	4.415 ± 0.015	3.55 ± 0.12	1.47 ± 0.01	5
